# Influence of adolescents’ and parental dietary knowledge on adolescents’ body mass index (BMI), overweight/obesity in 2004–2015: a longitudinal study

**DOI:** 10.1186/s13690-023-01197-x

**Published:** 2023-10-23

**Authors:** Zhengjie Cai, Ke Jiang, Tiankun Wang, Shengping Li, Jinli Xian, Yong Zhao, Zumin Shi

**Affiliations:** 1 School of Public Health, Chongqing Medical University, Chongqing, China; 2 West China School of Public Health and West China Fourth Hospital, Sichuan University, Chengdu, China; 3 Research Center for Medicine and Social Development, Chongqing Medical University, Chongqing, China; 4 Research Center for Public Health Security, Chongqing Medical University, Chongqing, China; 5 Department of Clinical Nutrition, West China Second Hospital, Sichuan University, Chengdu, China; 6 Key Laboratory of Birth Defects and Related Diseases of Women and Children, Ministry of Education, Chengdu, China; 7 Department of Children Healthcare, Women and Children’s Hospital of Chongqing Medical University, Chongqing, China; 8 The Department of clinical nutrition, Mianyang Central Hospital, Mianyang, China; 9 Chongqing Key Laboratory of Child Nutrition and Health, Children’s Hospital of Chongqing Medical University, Chongqing, China; 10 Human Nutrition Department, College of Health Sciences, QU Health, Qatar University, Doha, 2713 Qatar

**Keywords:** Adolescent, Dietary knowledge, Nutrition survey, Longitudinal study

## Abstract

**Objective:**

The global epidemic of overweight/obesity in children and adolescents poses a significant public health threat. This longitudinal study aims to investigate the relationship between adolescents’ and their parents’ dietary knowledge and overweight/obesity among adolescents in China.

**Methods:**

Data were collected from the China Health and Nutrition Survey (CHNS) in 2004, 2006, 2009, 2011 and 2015. Overweight/obesity was defined according to the International Obesity Task Force (IOTF) cut-off for body mass index (BMI). A set of questions were used to assess the dietary knowledge scores of both adolescents and their parents during face-to-face interviews. Mixed effect models were used to analyze the data.

**Results:**

A total of 2035 adolescents aged 12–17 years were included in the data analysis. The mean BMI increased from 19.2 in 2004 to 20.5 in 2015, with a significant increase in the prevalence of overweight and obesity from 6.8% and 0.5% in 2004 to 15.1% and 7.8% in 2015, respectively. Adolescents with medium-score dietary knowledge were less likely to be overweight/obese compared to those with low-score dietary knowledge (OR (95% CI): 0.20 (0.05–0.80), *P* < 0.05). However, there was no association between parental dietary knowledge and adolescents’ BMI or overweight/obesity (*P* > 0.05). Additionally, a significant interaction between adolescents’ dietary knowledge score and education in relation to adolescents’ BMI (*P* for interaction < 0.05).

**Conclusion:**

Adolescents with medium dietary knowledge were less likely to be overweight/obese than those with low knowledge, but no association was found with parental knowledge. Promoting nutritional education and healthy eating habits is vital to prevent overweight/obesity.

**Supplementary Information:**

The online version contains supplementary material available at 10.1186/s13690-023-01197-x.

**Table:** 

Text box 1. Contributions to the literature	
•This study provides evidence that adolescents with medium-score dietary knowledge are less likely to be overweight/obese and highlights the need for nutrition education programs that promote healthy eating habits.	
•The study also suggests that parental dietary knowledge may not be significantly associated with adolescents’ BMI or overweight/obesity.	
•The results underscore the importance of targeting adolescent nutrition education to prevent and address the increasing prevalence of overweight/obesity in this population.	
•Future research could explore the effectiveness of various implementation strategies in promoting healthy eating habits among adolescents.	

## Introduction

In recent years, overweight and obesity in children and adolescents have become a major public health concern globally due to the alarming increase in prevalence [1]. In China, the rapid economic development and changes in dietary patterns and lifestyles have led to a substantial increase in the prevalence of overweight and obesity in children and adolescents. The latest national estimates indicate a prevalence of 11.1% for overweight and 7.9% for obesity in children and adolescents aged 6–17 years in 2015–2019 [2, 3]. This represents a significant increase in prevalence compared to monitoring data in 1992, where prevalence was 3.9% for overweight and 1.8% for obesity [2, 3]. Childhood and adolescent overweight are likely to lead to lifelong obesity [4], and being overweight during this period is associated with a higher risk of chronic disorders, such as type 2 diabetes [5–7].

Effective prevention strategies of childhood obesity remain a public health priority [8]. Previous studies have shown that enhancing nutrition knowledge can help individuals adopt healthy eating and exercise habits to achieve energy balance [9, 10]. The World Health Organization’s Commission on Ending Childhood Obesity has also emphasized the importance of promoting nutrition knowledge among adolescents and parents or caregivers [11].Previous research has primarily focused on the relationship between nutrition or dietary knowledge and body mass index (BMI) in adults [12–14], with limited attention given to adolescents and their parents. Findings on the associations between dietary knowledge and BMI have been mixed. O’Dea et al. found no association between nutrition knowledge and BMI in Australian children and adolescents aged 6–18 years [15], while Said et al. reported a significant positive correlation between BMI z-scores and total dietary knowledge scores in Lebanese adolescents aged 15–18 years and their parents [16]. Conversely, Kakinami et al. observed an inverse association between nutrition knowledge and adiposity among Canadian children aged 10–15 years [17]. Similarly, a cross-sectional study in China also found that adolescents aged 8–18 years with high levels of dietary knowledge may be negatively associated with overweight and obesity, but it did not explore the effect of parental dietary knowledge on children’s overweight and obesity [18]. In another study, Subih et al. found no significant association between maternal dietary knowledge and mean BMI among children and adolescents aged 6–18 years in north Jordan, but a significantly decreased mean waist circumference among those whose mothers had a moderate and high level of nutritional knowledge compared with those whose mothers had a low level of nutritional knowledge [19]. Given the increasing prevalence of overweight and obesity among children and adolescents and the crucial role of parents in shaping their dietary habits and nutritional status, it is crucial to understand the impact of adolescents’ and parental dietary knowledge on adolescents’ BMI and overweight/obesity. However, limited studies have investigated this topic, and longitudinal evidence is still lacking. Therefore, this study aims to investigate the impact of dietary knowledge of Chinese adolescents aged 12–17 years and their parents on adolescents’ BMI and the prevalence of overweight and obesity, using longitudinal data from the China Health and Nutrition Survey (CHNS) conducted in 2004, 2006, 2009, 2011, and 2015.

## Methods and materials

### Study design and study sample

The China Health and Nutrition Survey (CHNS) is an ongoing international collaborative project designed to investigate the impact of social and economic changes on nutrition and health outcomes in China [20]. Surveys were conducted every 2–4 years. More details of the CHNS design, sampling and cohort profile information have been described in previous researches [21, 22]. The study was approved by the Institutional Review Boards of the University of North Carolina at Chapel Hill and the National Institute of Nutrition and Food Safety, China Center for Disease Control and Prevention. Each participant signed informed consent by their parents or caregivers.

**Fig. 1:**
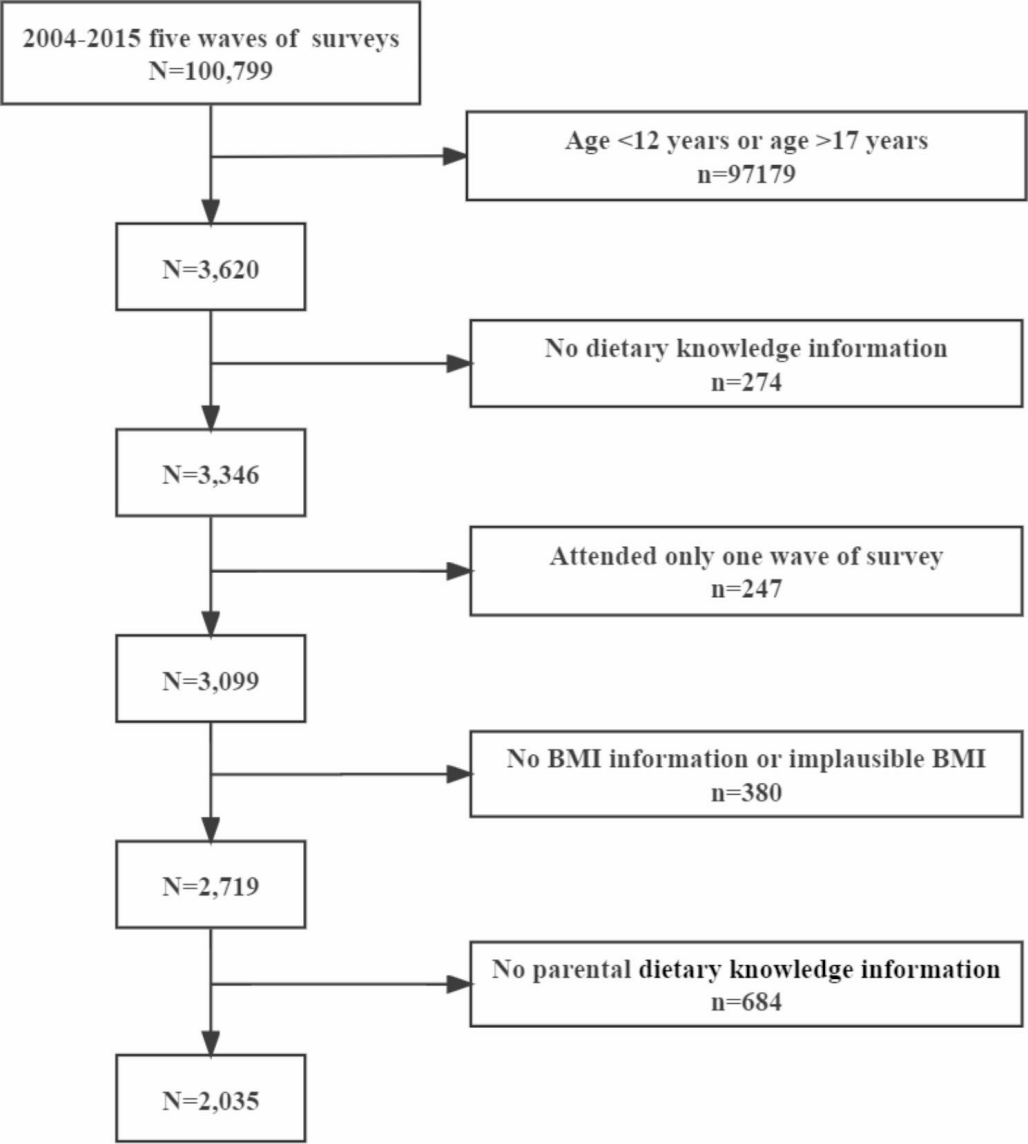
Participant flow chart

The dietary knowledge survey began in 2004, and available CHNS data from surveys conducted in 2004, 2006, 2009, 2011, and 2015 was utilized in this study. Individuals above the age of 12 were invited to answer the section on dietary knowledge. Hence, 3,620 adolescents aged 12–17 years participated in at least one of the five waves of surveys. Furthermore, we excluded adolescents who had no height and weight information or implausible BMI, those who had no dietary knowledge information, and whose parents had no dietary knowledge information. Finally, 2,035 adolescents were included in our data analysis (Fig. 1), numbers of adolescents extracted were 630 (2004), 423 (2006), 367 (2009), 410 (2011), 205 (2015).

### Outcome variables: BMI and overweight/obesity

The weight and height of adolescents were measured by at least two trained health professionals who followed standard protocol and techniques. One professional took the measurements, while another recorded the reading data. Weight was measured in light indoor clothing without shoes to the nearest tenth of a kilogram using a beam balance scale, and height was measured without shoes to the nearest tenth of a centimeter using a portable stadiometer [23]. BMI, defined as the body weight in kilograms divided by the squared body height in meters, is used here as the indicator of adolescents’ overweight and obesity. Moreover, overweight and obesity were defined based on the International Obesity Task Force (IOTF) recommended age-sex-specific BMI cut-off-points [24].

### Exposure variables: dietary knowledge of adolescents and their parents

The description of 17 questions in terms of dietary knowledge is presented in Supplementary Tables 1, which includes 12 questions from the 2004, 2006, 2009, and 2011 surveys and five additional questions added in 2015. The five new questions added in 2015 were: “eating salty foods can cause hypertension”, “refined grains (rice and wheat flour) contain more vitamins and minerals than unrefined grains”, “lard is healthier than vegetable oils”, “vegetables contain more starch than staple foods (rice or wheat flour)”, “eggs and milk are the important sources of high-quality protein”. And these 17 questions have been validated in previous studies [14, 18]. Responses to the dietary knowledge questions were categorized as correct, wrong, neutral, or unknown, with a score of 1 assigned for a correct answer and 0 for a wrong, neutral, or unknown answer. To ensure comparability, we transformed the scores into percentages for each survey wave, and then divided the scores into tertiles for both adolescents and their parents for the analysis.

### Covariates

Adolescents’ age, sex (boys/girls), residence (urban/rural areas), per capita annual family income (tertiles: low, medium, and high), education level of adolescents and their parents (low: primary school or below; medium: secondary school/secondary vocational school; high: high school or above), overweight and obesity status of adolescents’ parents (no/yes), physical activity (metabolic equivalent of task (MET), h /week), parental diagnosed hypertension (no/yes), and parental diagnosed diabetes (no/yes), were considered as covariates in this study. And the classifications of these variables were based on previous studies [25, 26].

### Statistical analysis

Descriptive statistics were used to summarize the sample characteristics. Categorical variables were described using frequencies and percentages, while the mean (standard deviation) was used to describe adolescents’ and their parents’ BMI. Missing values of the covariates were imputed using regression imputation with the ‘mi impute regress’ command in Stata [27, 28]. We used the chi-square test for categorical variables and ANOVA for continuous variables to compare differences between groups. Then, we used mixed effect models in Stata to assess the association between adolescents’ and parental dietary knowledge and adolescents’ BMI and overweight/obesity adjusting for covariates. In addition, we conducted a subgroup analysis of the associations between adolescents’ and parental dietary knowledge and adolescent’ BMI and overweight/obesity by sociodemographic characteristics. All tests were two-sided, and *P-value* < 0.05 was considered to be statistically significant. All the analyses were performed using STATA 15.1 (Stata Corporation, College Station).

## Results

### Characteristics of the study sample

Table 1a and Table 1b present the descriptive statistics for Chinese adolescents aged 12–17 years in the CHNS across tertile of both adolescents’ and their parental dietary knowledge scores. Our study included 2,035 adolescents, of whom 54.7% were aged 12–14 years, 53.6% were boys, and 68.4% resided in rural areas. The majority (63.4%) of the adolescents attended secondary school/secondary vocational school, and approximately half of their fathers (45.2%) and mothers (43.0%) had a medium level of education. Moreover, 11.6% of the adolescents were classified as overweight or obese, while 42.6% and 36.8% of their fathers and mothers, respectively, were classified as overweight or obese. Besides, we observed no significant difference in the overweight/obesity rates across tertiles of the adolescents’ dietary knowledge scores (P = 0.05), and observed that adolescents’ BMI was significantly higher when their parents’ dietary knowledge scores were in the high group.

**Table 1a:** Sample characteristics by tertiles of adolescents’ dietary knowledge score: CHNS (N = 2035)

Characteristics	Total	Adolescents’ dietary knowledge score			*P*-value
		Tertile 1	Tertile 2	Tertile 3	
Sample (n)	N = 2035	N = 485	N = 702	N = 848	
Age (n, %)					0.01^*^
12–14	1113 (54.7)	296 (61.0)	371 (52.8)	446 (52.6)	
15–17	922 (45.3)	189 (39.0)	331 (47.2)	402 (47.4)	
Sex (n, %)					0.09
Boys	1,091 (53.6)	277 (57.1)	381 (54.3)	433 (51.1)	
Girls	944 (46.4)	208 (42.9)	321 (45.7)	415 (48.9)	
Residence (n, %)					< 0.01^**^
Rural	1,391 (68.4)	349 (72.0)	470 (67.0)	572 (67.5)	0.14
Urban	644 (31.6)	136 (28.0)	232 (33.0)	276 (32.5)	
Family income (n, %)					0.25
Low	405 (19.9)	100 (20.6)	147 (20.9)	158 (18.6)	
Medium	696 (34.2)	178 (36.7)	241 (34.3)	277 (32.7)	
High	934 (45.9)	207 (42.7)	314 (44.7)	413 (48.7)	
Adolescents’ education level (n, %)					< 0.01^**^
Low	292 (14.3)	97 (20.0)	90 (12.8)	105 (12.4)	
Medium	1291 (63.4)	303 (62.5)	453 (64.5)	535 (63.1)	
High	452 (22.2)	85 (17.5)	159 (22.6)	208 (24.5)	
Father’s education level (n, %)					0.06
Low	412 (20.2)	116 (23.9)	135 (19.2)	161 (19.0)	
Medium	919 (45.2)	222 (45.8)	325 (46.3)	372 (43.9)	
High	704 (34.6)	147 (30.3)	242 (34.5)	315 (37.1)	
Mother’s education level (n, %)					0.03^*^
Low	619 (30.4)	168 (34.6)	196 (27.9)	255 (30.1)	
Medium	875 (43.0)	210 (43.3)	316 (45.0)	349 (41.2)	
High	541 (26.6)	107 (22.1)	190 (27.1)	244 (28.8)	
Survey year (n, %)					< 0.01^**^
2004	630 (31.0)	97 (20.0)	216 (30.8)	317 (37.4)	
2006	423 (20.8)	104 (21.4)	175 (24.9)	144 (17.0)	
2009	367 (18.0)	91 (18.8)	129 (18.4)	147 (17.3)	
2011	410 (20.1)	89 (18.4)	118 (16.8)	203 (23.9)	
2015	205 (10.1)	104 (21.4)	64( 9.1)	37(4.4)	
Adolescents’ BMI (kg/m^2^), mean (SD)	19.5 (3.3)	19.4 (3.6)	19.5(3.2)	19.5(3.2)	0.77
Adolescents’ BMI category (n, %)					0.05
Not overweight or obese	1,799 (88.4)	414 (85.4)	629 (89.6)	756 (89.2)	
Overweight or obese	236 (11.6)	71 (14.6)	73 (10.4)	92 (10.8)	
Father’s BMI (kg/m^2^), mean (SD)	23.7 (3.2)	23.7(3.2)	23.7(3.1)	23.7 (3.3)	0.96
Father’s BMI category (n, %)					0.83
Not overweight or obese	1169 (57.4)	283 (58.4)	405 (57.7)	481 (56.7)	
Overweight or obese	866 (42.6)	202 (41.6)	297 (42.3)	367 (43.3)	
Mother’s BMI (kg/m^2^), mean (SD)	23.3 (3.2)	23.3 (3.2)	23.2 (3.2)	23.3 (3.2)	0.85
Mother’s BMI category (n, %)					0.96
Not overweight or obese	1286 (63.2)	307 (63.3)	446 (63.5)	533 (62.9)	
Overweight or obese	749 (36.8)	178 (36.7)	256 (36.5)	315 (37.1)	

Data are presented as mean (SD) for continuous measures, and n (%) for categorical measures

* *P* < 0.05; ** *P* < 0.01

**Table 1b:** Sample characteristics by tertiles of parental dietary knowledge score: CHNS (N = 2035)

Characteristics	Fathers’ dietary knowledge score			*P*-value	Mothers’ dietary knowledge score			*P*-value
	Tertile 1	Tertile 2	Tertile 3		Tertile 1	Tertile 2	Tertile 3	
Sample (n)	N = 415	N = 659	N = 961		N = 410	N = 704	N = 921	
Age (n, %)				0.13				0.07
12–14	245 (59.0)	357 (54.2)	511 (53.2)		239 (58.3)	395 (56.1)	479 (52.0)	
15–17	170 (41.0)	302 (45.8)	450 (46.8)		171 (41.7)	309 (43.9)	442 (48.0)	
Sex (n, %)				0.22				0.85
Boys	234 (56.4)	337 (51.1)	520 (54.1)		224 (54.6)	379 (53.8)	488 (53.0)	
Girls	181 (43.6)	322 (48.9)	441 (45.9)		186 (45.4)	325 (46.2)	433 (47.0)	
Residence (n, %)				< 0.01^**^				< 0.01^**^
Rural	318 (76.6)	435 (66.0)	638 (66.4)		313 (76.3)	491 (69.7)	587 (63.7)	
Urban	97 (23.4)	224 (34.0)	323 (33.6)		97 (23.7)	213 (30.3)	334 (36.3)	
Family income (n, %)				< 0.01^**^				< 0.01^**^
Low	112 (27.0)	132 (20.0)	161 (16.8)		110 (26.8)	145 (20.6)	150 (16.3)	
Medium	119 (28.7)	252 (38.2)	325 (33.8)		141 (34.4)	249 (35.4)	306 (33.2)	
High	184 (44.3)	275 (41.7)	475 (49.4)		159 (38.8)	310 (44.0)	465 (50.5)	
Adolescents’ education level (n, %)				< 0.01^**^				< 0.01^**^
Low	80 (19.3)	72 (10.9)	140 (14.6)		73 (17.8)	109 (15.5)	110 (11.9)	
Medium	258 (62.2)	453 (68.7)	580 (60.4)		260 (63.4)	452 (64.2)	579 (62.9)	
High	77 (18.6)	134 (20.3)	241 (25.1)		77 (18.8)	143 (20.3)	232 (25.2)	
Father’s education level (n, %)				< 0.01^**^				< 0.01^**^
Low	112 (27.0)	126 (19.1)	174 (18.1)		111 (27.1)	141 (20.0)	160 (17.4)	
Medium	199 (48.0)	314 (47.6)	406 (42.2)		184 (44.9)	334 (47.4)	401 (43.5)	
High	104 (25.1)	219 (33.2)	381 (39.6)		115 (28.0)	229 (32.5)	360 (39.1)	
Mother’s education level (n, %)				< 0.01^**^				< 0.01^**^
Low	159 (38.3)	191 (29.0)	269 (28.0)		170 (41.5)	222 (31.5)	227 (24.6)	
Medium	183 (44.1)	285 (43.2)	407 (42.4)		171 (41.7)	307 (43.6)	397 (43.1)	
High	73 (17.6)	183 (27.8)	285 (29.7)		69 (16.8)	175 (24.9)	297 (32.2)	
Survey year (n, %)				< 0.01^**^				< 0.01^**^
2004	92 (22.2)	204 (31.0)	334 (34.8)		96 (23.4)	205 (29.1)	329 (35.7)	
2006	101 (24.3)	148 (22.5)	174 (18.1)		87 (21.2)	165 (23.4)	171 (18.6)	
2009	64 (15.4)	102 (15.5)	201 (20.9)		75 (18.3)	126 (17.9)	166 (18.0)	
2011	64 (15.4)	134 (20.3)	212 (22.1)		68 (16.6)	130 (18.5)	212 (23.0)	
2015	94 (22.7)	71 (10.8)	40( 4.2)		84 (20.5)	78 (11.1)	43 (4.7)	
Adolescents’ BMI (kg/m^2^), mean (SD)	19.4(3.6)	19.2 (3.0)	19.7(3.2)	0.03^*^	19.2 (3.4)	19.3(3.2)	19.7(3.3)	0.01^*^
Adolescents’ BMI category (n, %)				0.33				0.06
Not overweight or obese	367 (88.4)	592 (89.8)	840 (87.4)		365 (89.0)	636 (90.3)	798 (86.6)	
Overweight or obese	48 (11.6)	67 (10.2)	121 (12.6)		45 (11.0)	68( 9.7)	123 (13.4)	
Father’s BMI (kg/m^2^), mean (SD)	23.3(3.3)	23.7 (3.1)	23.9 (3.1)	0.01^*^	23.4(3.3)	23.6(3.1)	23.9(3.2)	0.03^*^
Father’s BMI category (n, %)				0.03^*^				0.28
Not overweight or obese	261 (62.9)	376 (57.1)	532 (55.4)		245 (59.8)	412 (58.5)	512 (55.6)	
Overweight or obese	154 (37.1)	283 (42.9)	429 (44.6)		165 (40.2)	292 (41.5)	409 (44.4)	
Mother’s BMI (kg/m^2^), mean (SD)	23.2 (3.4)	23.2 (3.0)	23.4 (3.2)	0.29	23.3 (3.3)	23.2 (3.2)	23.4 (3.1)	0.45
Mother’s BMI category (n, %)				0.47				0.73
Not overweight or obese	267 (64.3)	425 (64.5)	594 (61.8)		256 (62.4)	453 (64.3)	577 (62.6)	
Overweight or obese	148 (35.7)	234 (35.5)	367 (38.2)		154 (37.6)	251 (35.7)	344 (37.4)	

Data are presented as mean (SD) for continuous measures, and n (%) for categorical measures

* *P* < 0.05; ** *P* < 0.01

### The trend in BMI, BMI categories of adolescents and adolescents’ and parental dietary knowledge score by survey year

**Fig. 2:**
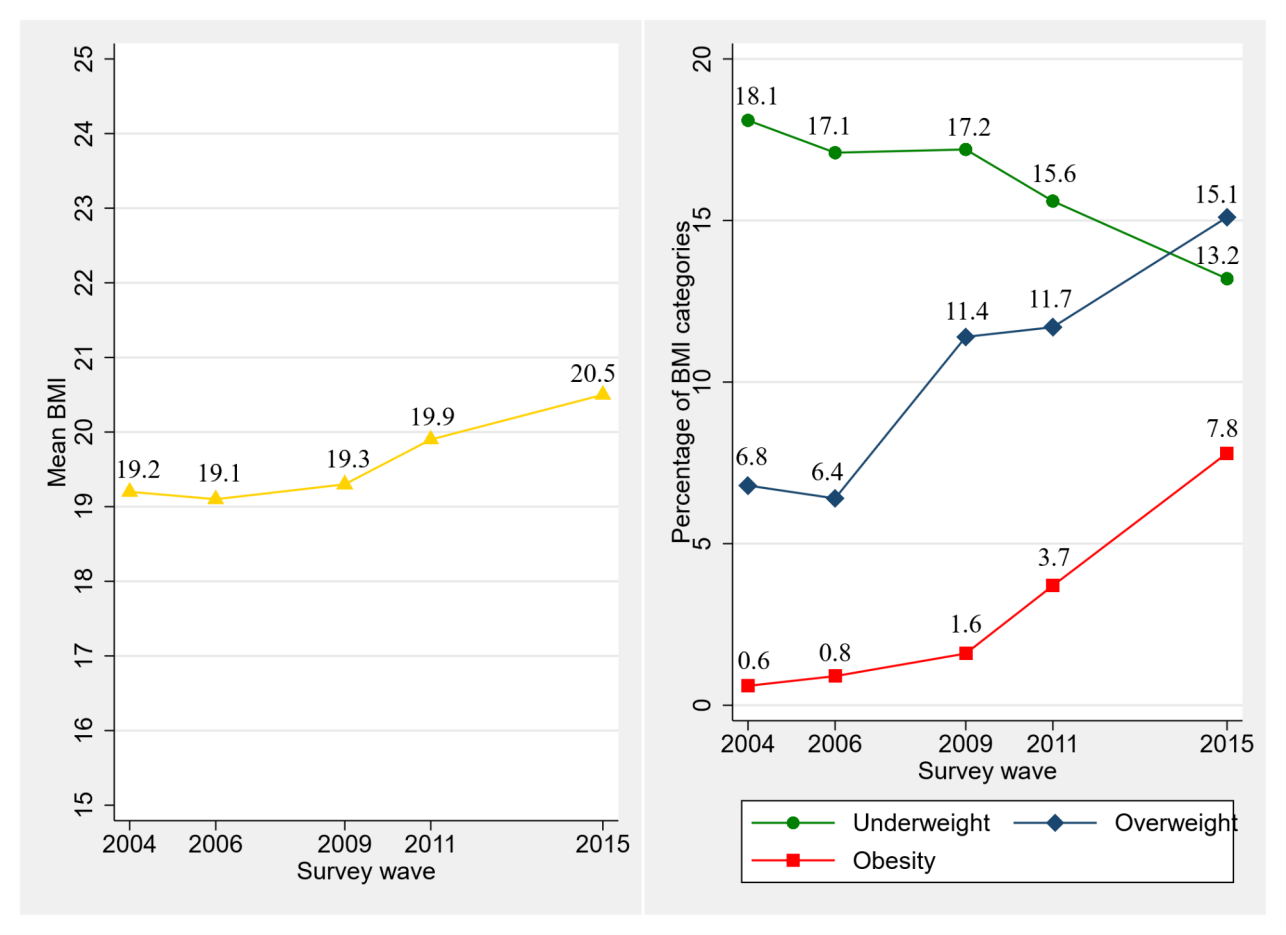
Trend in BMI and BMI categories of adolescents (CHNS 2004, 2006, 2009, 2011, 2015)

Figure 2 displays a significant increase in the BMI of Chinese adolescents in our study from 2004 to 2015. Mean BMI increased from 19.2 kg/m^2^ to 20.5 kg/m^2^ over the survey period, with the prevalence of overweight more than doubling from 6.8% to 15.1% and the prevalence of obesity increasing approximately thirteen times from 0.6% to 7.8%. In contrast, the proportion of underweight adolescents decreased from 18.1% to 13.2%.

**Fig. 3:**
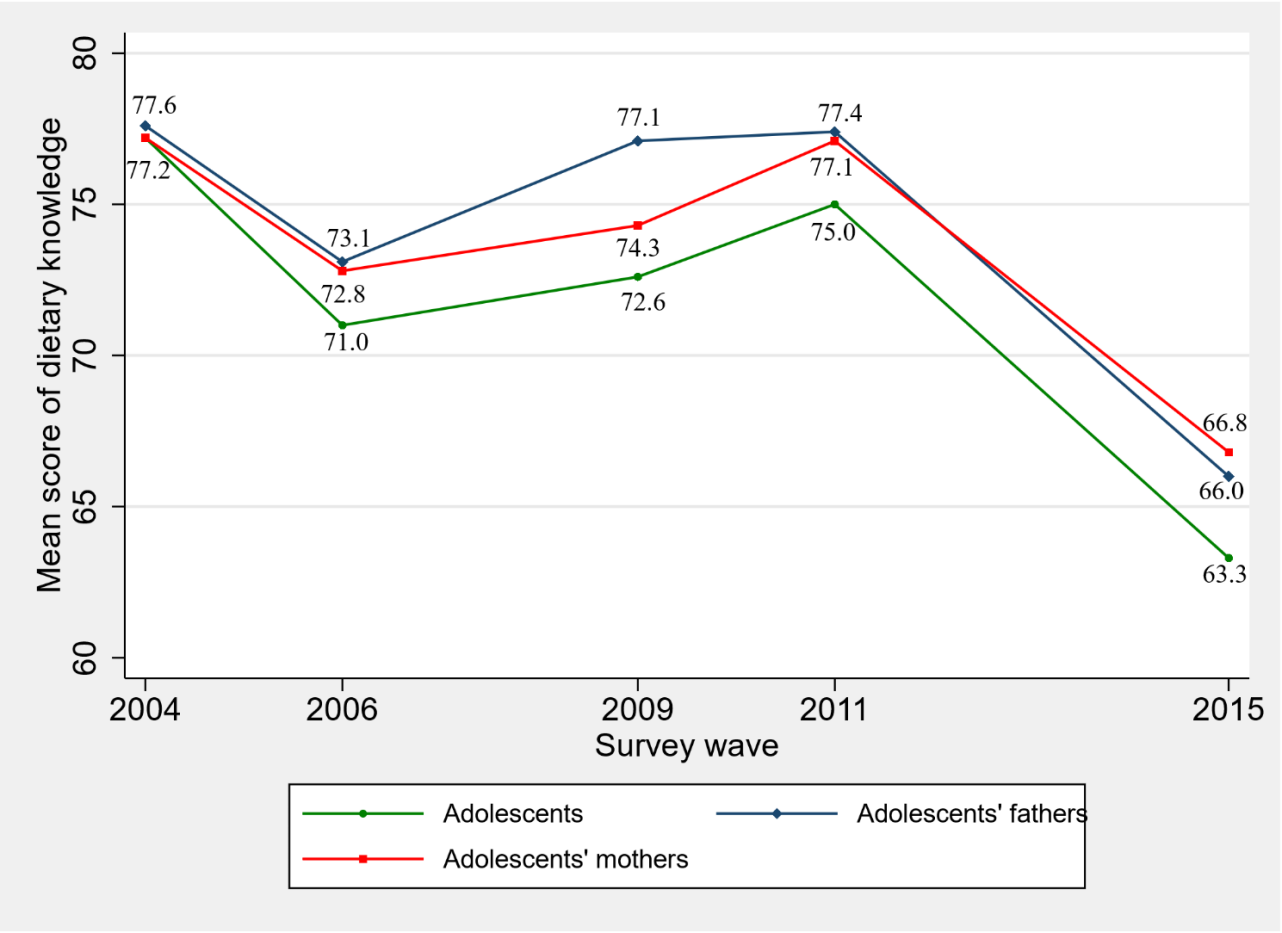
Trend in adolescents’ and parental dietary knowledge score (CHNS 2004, 2006, 2009, 2011, 2015)

Figure 3 illustrates the mean scores of adolescents’ and their parents’ dietary knowledge from 2004 to 2015. The mean score of adolescents’ dietary knowledge was 77.2, 71.0, 72.6, and 75.0 in 2004, 2006, 2009, and 2011, respectively, while the mean score of parents’ dietary knowledge was 77.6, 73.1, 77.1, and 77.4 for fathers and 77.2, 72.8, 74.3, and 77.1 for mothers during the same period. However, the lowest scores were observed in 2015, with adolescents and their parents achieving mean scores of 63.3, 66.0, and 66.8, respectively.

### Associations between adolescents’ and parental dietary knowledge score and BMI and overweight/obesity of adolescents

Associations between adolescents’ and parental dietary knowledge score and BMI and overweight/obesity of adolescents are presented in Table 2. After adjusting for covariates, compared with low-score group, adolescents with medium-score dietary knowledge were less likely to be overweight/obesity (OR: 0.20, 95% CI: 0.05–0.80), *P* < 0.05). However, there were no significant differences between parental dietary knowledge score, and BMI or overweight/obesity status of adolescents (*P* > 0.05).

**Table 2:** Mixed effect models on the associations between adolescents’ and parental dietary knowledge score and overweight/obesity of adolescents aged 12–17 years old

Variables	Adjusted model ^a^ (BMI as categorical variable)		Adjusted model ^a^ (BMI as continuous variable)	
	OR (95% CI)	*P*-value	β (95% CI)	*P*-value
Dietary knowledge score of adolescents				
Medium vs. low	**0.20 (0.05–0.80)**	**0.02** ^*^	-0.09 (-0.40–0.23)	0.59
High vs. low	0.47 (0.15–1.50)	0.20	-0.29 (-0.33–0.27)	0.85
Dietary knowledge score of adolescents’ fathers				
Medium vs. low	0.50 (0.12–2.04)	0.34	-0.24 (-0.56–0.09)	0.15
High vs. low	0.21 (0.05–0.99	0.06	0.01 (-0.29–0.32)	0.93
Dietary knowledge score of adolescents’ mothers				
Medium vs. low	0.34 (0.08–1.40)	0.14	-0.17 (-0.49–0.15)	0.31
High vs. low	0.45 (0.12–1.64)	0.23	0.03 (-0.29–0.34)	0.85

^a^ Adjusted for adolescents’ age and sex, residence, per capita annual family income, physical activity level, adolescents’ education and parental education, parental hypertension and diabetes and parental overweight/obesity status

* *P* < 0.05

### Subgroup analysis of the associations between adolescents’ dietary knowledge score and BMI and overweight/obesity

No significant interactions were observed between adolescents’ dietary knowledge score and sex, residence, or per capita annual family income for their overweight/obesity status (Table 3). Similarly, no significant interactions were observed between adolescents’ dietary knowledge score and sex, residence, or per capita annual family income for their BMI. However, a significant interaction was observed between adolescents’ dietary knowledge score and education. Among adolescents with high school education or above, a high dietary knowledge score was negatively associated with BMI (*P* for interaction <0.05). However, no statistically significant association was found between participants with low or medium levels of education and adolescent’s BMI.

**Table 3:** Subgroup analysis of the associations between adolescents’ dietary knowledge score and BMI and overweight/obesity: CHNS (2004–2015)

Variables	BMI as a categorical variable				BMI as a continuous variable			*P* for interaction
	Adolescents’ dietary knowledge score ((OR (95%CI))			*P* for interaction	Adolescents’ dietary knowledge score (β (95%CI))			
	Low	Medium	High		Low	Medium	High	
Sex				0.42				0.83
Boys	1.00	0.69 (0.41–1.15)	**0.50 (0.29–0.87)**		0.00	-0.12 (-0.57–0.31)	-0.02(-0.40–0.44)	
Girls	1.00	0.65 (0.33–1.30)	0.77 (0.40–1.49)		0.00	0.06(-0.50–0.37)	-0.05 (-0.47–0.36)	
Residence				0.45				0.20
Rural	1.00	0.66 (0.40–1.11)	0.72 (0.43–1.23)		0.00	0.08 (-0.30–0.45)	0.14 (-0.22–0.50)	
Urban	1.00	0.64 (0.32–1.28)	**0.42 (0.20–0.87)**		0.00	-**0.60 (-1.14–-0.06)**	-0.45 (-0.98–0.07)	
Family income				0.56				0.20
Low	1.00	0.82 (0.26–2.60)	1.38 (0.42–4.51)		0.00	**0.61 (0.01–1.21)**	**0.62 (0.03–1.23)**	
Medium	1.00	0.42 (0.20–0.90)	**0.35 (0.16–0.76)**		0.00	-0.31 (-0.76–0.15)	-0.20 (-0.63–0.22)	
High	1.00	0.84 (0.48–1.48)	0.68 (0.38–1.21)		0.00	-0.02 (-0.58–0.56)	-0.19 (-0.73–0.35)	
Adolescents’ education				0.46				0.04^*^
Low	1.00	**0.22 (0.07–0.69**)	**0.18 (0.06–0.58)**		0.00	-0.26 (-1.19–0.66)	0.00 (-0.86–0.85)	
Medium	1.00	0.90 (0.53–1.54)	0.90 (0.52–1.55)		0.00	0.09 (-0.30–0.48)	0.10 (-0.27–0.48)	
High	1.00	0.58 (0.23–1.44)	**0.38 (0.15–0.98)**		0.00	**-0.81 (-1.56–--0.05)**	**-1.05 (-1.78–-0.33)**	

Results in bold are statistically significant (*P* < 0.05)

^a^ Adjusted for adolescents’ age and sex, residence, per capita annual family income, physical activity level, adolescents’ education and parental education, parental hypertension and diabetes and parental overweight/obesity status

* *P* < 0.05

## Discussion

This prospective cohort study found an inverse association between Chinese adolescents’ dietary knowledge and their overweight/obesity status but not with BMI. However, no statistically significant association was found between parental dietary knowledge and adolescents’ BMI or overweight/obesity. Improving the level of adolescents’ dietary knowledge can be considered an important measure for preventing and controlling overweight/obesity.

To the best of our knowledge, this is the first longitudinal study that examines the associations between adolescents’ and parental dietary knowledge and adolescents’ BMI and overweight/obesity. The current study found that adolescents with medium-score dietary knowledge were less likely to be overweight/obesity compared those with low dietary knowledge score. The results were similar with previous cross-sectional studies among children and adolescents in Turkey [29], China [30], which reported a significant reduction in the odds ratio of obesity with increasing levels of nutrition knowledge. Improving nutrition knowledge in children and adolescents may help promote healthy dietary habits and intake [31, 32]. Dietary behaviors play a crucial role in determining the obesity risk of children and adolescents [33]. Thus, it is possible that the higher level of dietary knowledge among adolescents in the present study was associated with a greater adherence to healthy dietary behaviors, which in turn resulted in a lower risk of overweight/obesity. Future studies should examine the mediating role of dietary behaviors between dietary knowledge and overweight/obesity in Chinese adolescents. However, we found that high dietary knowledge scores in adolescents did not necessarily lead to a reduced risk of overweight and obesity. While possessing knowledge on healthy dietary behaviors is essential, it does not necessarily translate to consistent healthy eating habits. Adolescents may still consume high-calorie foods and have a lack of physical activity, leading to an increased risk of overweight and obesity, despite their awareness of healthy eating [34]. Conversely, adolescents with moderate dietary knowledge may not have fully mastered how to achieve a healthy diet. As a result, they may display a greater motivation to strictly control their diet, in order to reduce the risk of overweight and obesity. This finding might suggest that achieving a healthy diet is not solely dependent on having high levels of dietary knowledge but also on adopting healthy eating behaviors consistently [33].Also, some studies targeting children have reported no significant correlation between nutrition or dietary knowledge levels and nutritional status [15, 35]. Age-related differences may contribute to the contradictory findings in previous studies. For younger children, parents or caregivers play a significant role in helping children develop healthy behaviors and are responsible for daily diet decisions, making adolescents’ dietary knowledge less influential. This explanation could apply to our another finding that parental dietary knowledge was not significantly associated with adolescents’ BMI or overweight/obesity status. The age of investigated adolescents was 12–17 years old in this study, and they are experiencing the period of adolescent rebellion and might have been less compliant to parents or caregivers, and they develop independent thoughts when it comes to eating behaviors [36]. Story et al. mentioned that as adolescents go through significant changes during this period, such as growing independence and eating away from home, parents have less control over what their children eat [37]. Additionally, Asakura et al. pointed out that communication between caregivers and children about nutrition and foods might be insufficient, thus, the nutrition knowledge of caregivers may have a slight effect on the dietary intake of their children [38], not to mention those adolescents who were boarding at school. Williams et al. demonstrated that parents with high dietary knowledge may provide children and adolescents with a healthy diet, but adolescents might be able to alter a variety of foods offered from their parents frequently by refusing foods they would not like to consume [39]. And Räsänen et al. reported that when a nutrition counseling intervention was given to parents, the nutrition knowledge score of children was not improved in the intervention group compared with the control group [40]. Moreover, in many countries, especially in China, due to the traditional family structure of three-generation households, grandparental child care is more prevalent and plays a significant role in child care [41, 42]. As grandparents assume the role of parents in children’s lives, they may exert more influence over children’s dietary behaviors and nutritional status compared to parents. Therefore, further studies should explore the impact of grandparents’ dietary knowledge on the nutritional status of children and adolescents. These findings suggest that food education programs targeting adolescents may be more effective in improving their nutritional status than programs aimed at parents in China.

Our study found a significant interaction between adolescents’ dietary knowledge score and education in relation to adolescents’ BMI. Specifically, among adolescents with high school education or above, a high dietary knowledge score was negatively associated with BMI. However, no such association was found in participants with low or medium levels of education. These results suggest that adolescents with higher levels of education may be more likely to consistently apply their dietary knowledge in practice. Thus, educational level should be taken into consideration when analyzing the association between dietary knowledge and adolescents’ nutritional status.

The prevalence of overweight and obesity among Chinese adolescents is a critical issue emphasized in our study. Yaru et al. reported an overall upward trend of overweight/obesity among Chinese children and adolescents from 1991 to 2015 in a recent meta-analysis [43]. Our results confirmed that overweight/obesity of adolescents are becoming an increasingly serious matter in China. The prevalence of overweight has increased from 6.8% in 2004 to 15.1% in 2015, and the prevalence of obesity remarkably increased approximately thirteen times from 0.6% in 2004 to 7.8% in 2015. Some measures, such as improving the level of adolescents’ dietary knowledge according to the results of this study should be considered. In addition, it is worth noting that adolescents’ dietary knowledge score are 77.2, 71.0, 72.6, 75.0 in 2004, 2006, 2009 and 2011, respectively, while it dropped to 63.3 in 2015. The different difficulty degree between five new questions added in the survey of 2015 and the questions in previous surveys may be one possible reason. More importantly, it reflects that dietary knowledge of Chinese adolescents needs to be improved over recent years in China. However, dietary education is not yet a part of the curriculum for Chinese students leaving few scientific and rational channels for students to obtain accurate and systematic dietary knowledge [13]. Systematic dietary nutrition education is urgently needed, in China and what is more importantly is that incorporating dietary/nutrition courses into the current compulsory education system, and consequently, decreasing the overweight/obesity rate of Chinese children and adolescents.

The results from the present study should be interpreted with caution as there are some limitations. Firstly, we only measured general dietary knowledge, which may not fully reflect the entire spectrum of dietary knowledge. Thus, there may be some biases in accurately and objectively assessing the level of adolescents’ and parental dietary knowledge. Secondly, the use of self-reported data may introduce biases caused by dishonesty and measurement flaws. Thirdly, we were unable to consider the impact of confounding factors such as TV programs, applications or video games on our results, due to the lack of relevant data in the CHNS. Despite these limitations, our study addresses the existing gap in the literature regarding the influence of adolescents’ and parents’ dietary knowledge on adolescents’ weight status and aims to contribute to the effective prevention and control of obesity among this population. Additionally, the longitudinal data provided stronger evidence than a cross-sectional study.

## Conclusion

Our study confirms the increasing seriousness of overweight and obesity among Chinese adolescents aged 12–17 years. The results of our mixed-effect models suggest that adolescents with medium-score dietary knowledge were less likely to be overweight/obese compared to those with low-score dietary knowledge, while no significant association was found between parental dietary knowledge and adolescents’ BMI or overweight/obesity. Our findings highlight the need to promote nutrition education programs that improve dietary knowledge and healthy eating habits among adolescents, as a preventative measure for overweight/obesity.

### Electronic supplementary material

Below is the link to the electronic supplementary material.

Supplementary Material 1

## Data Availability

The datasets generated during and/or analyzed during the currentstudy are available in the CHNS repository, https://www.cpc.unc.edu/projects/china (accessed on 24 March 2023).

## Abbreviations

BMI: Body mass index
CHNS: China Health and Nutrition Survey
IOTF: International Obesity Task Force

## References

[1] WHO. (2018). Taking action on childhood obesity, World Health Organization. https://apps.who.int/iris/handle/10665/274792. Accessed 24 December 2022.

[2] Pan XF, Wang L, Pan A (2021). Epidemiology and determinants of obesity in China. Lancet Diabetes Endocrinol.

[3] The report on chronic disease and nutrition of Chinese residents. (2020) (2020). The report on chronic disease and nutrition of Chinese residents (2020).: National Health Commission of the People’s Republic of China.; 2020. http://www.gov.cn/xinwen/2020-12/23/content_5572785.htm.Accessed.

[4] Singh AS, Mulder C, Twisk JW, van Mechelen W, Chinapaw MJ (2008). Tracking of childhood overweight into adulthood: a systematic review of the literature. OBES REV.

[5] WHO. Consideration of the evidence on childhood obesity for the commission on ending childhood obesity: report of the ad hoc working group on science and evidence for ending childhood obesity, Geneva, Switzerland. World Health Organization; 2016. https://apps.who.int/iris/handle/10665/206549. Accessed 21 December 2022.

[6] Kelishadi R, Haghdoost A, Sadeghirad B, Khajehkazemi R (2014). Trend in the prevalence of obesity and overweight among iranian children and adolescents: a systematic review and meta-analysis. NUTRITION.

[7] Twig G, Yaniv G, Levine H, Leiba A, Goldberger N, Derazne E, et al. Body-Mass Index in 2.3 million Adolescents and Cardiovascular Death in Adulthood. N Engl J Med. 2016;374. 10.1056/NEJMoa1503840. 10.1056/NEJMoa1503840 27074389

[8] Anderson KL (2018). A review of the Prevention and Medical Management of Childhood obesity. Child Adolesc Psychiatr Clin N Am.

[9] Bonaccio M, Di Castelnuovo A, Costanzo S, De Lucia F, Olivieri M, Donati MB et al. (2013). Nutrition knowledge is associated with higher adherence to Mediterranean diet and lower prevalence of obesity. Results from the Moli-sani study. APPETITE.;68:139 – 46. 10.1016/j.appet.2013.04.026. 10.1016/j.appet.2013.04.026 23665233

[10] Wagner M, Rhee Y, Hert-Honrath K, Salafia E, Terbizan D. Nutrition education effective in increasing fruit and vegetable consumption among overweight and obese adults. APPETITE. 2016;100. 10.1016/j.appet.2016.02.002. 10.1016/j.appet.2016.02.002 26850310

[11] WHO. (2016). The Report of the Commission on Ending Childhood Obesity.https://www.who.int/end-childhood-obesity/en/. Accessed 10 December 2022.

[12] Akkartal ^, Gezer C (2020). Is Nutrition Knowledge related to Diet Quality and obesity?. ECOL FOOD NUTR.

[13] Zhou L, Zeng Q, Jin S, Cheng G (2017). The impact of changes in dietary knowledge on adult overweight and obesity in China. PLoS ONE.

[14] Yu J, Han X, Wen H, Ren J, Qi L. (2020). Better Dietary Knowledge and Socioeconomic Status (SES), Better Body Mass Index? Evidence from China-An Unconditional Quantile Regression Approach. NUTRIENTS.;12(4). 10.3390/nu12041197. 10.3390/nu12041197 PMC7231000 32344738

[15] O’Dea JA, Wilson R (2006). Socio-cognitive and nutritional factors associated with body mass index in children and adolescents: possibilities for childhood obesity prevention. HEALTH EDUC RES.

[16] Said L, Gubbels JS, Kremers S. (2020). Dietary knowledge, Dietary Adherence, and BMI of lebanese adolescents and their parents. NUTRIENTS.;12(8). 10.3390/nu12082398. 10.3390/nu12082398 PMC7468749 32796513

[17] Kakinami L, Houle-Johnson S, McGrath JJ (2016). Parental Nutrition Knowledge Rather Than Nutrition label use is Associated with Adiposity in Children. J NUTR EDUC BEHAV.

[18] Wang L, Zhuang J, Zhang H, Lu W (2022). Association between dietary knowledge and overweight/obesity in chinese children and adolescents aged 8–18 years: a cross-sectional study. BMC PEDIATR.

[19] Subih HS, Abu-Shquier Y, Bawadi H, Al-Bayyari N (2018). Assessment of body weight, maternal dietary knowledge and lifestyle practices among children and adolescents in north Jordan. PUBLIC HEALTH NUTR.

[20] China Health and Nutrition Survey China Health. and Nutrition Survey.https://www.cpc.unc.edu/projects/china. Accessed.

[21] Zhang B, Zhai FY, Du SF, Popkin BM. (2014). The China Health and Nutrition Survey, 1989–2011. OBES REV.;15 suppl 1(0 1):2–7. 10.1111/obr.12119. 10.1111/obr.12119 PMC3869031 24341753

[22] Popkin BM, Du S, Zhai F, Zhang B (2009). Cohort Profile: the China Health and Nutrition Survey—monitoring and understanding socio-economic and health change in China, 1989–2011. INT J EPIDEMIOL.

[23] Popkin BM, Paeratakul S, Zhai F, Ge K. (1995). Dietary and Environmental Correlates of Obesity in a Population Study in China. Obesity Research.;3(S2):135s-143s. 10.1002/j.1550-8528.1995.tb00456.x. 10.1002/j.1550-8528.1995.tb00456.x 8581769

[24] Cole TJ, Bellizzi MC, Flegal KM, Dietz WH (2000). Establishing a standard definition for child overweight and obesity worldwide: international survey. BMJ (Clinical Research ed.

[25] Xian J, Zeng M, Cai Z, Xie C, Xie Y, Sharma M (2021). Influence of the request and purchase of television advertised foods on dietary intake and obesity among children in China. BMC Public Health.

[26] Huang L, Lyu J, Long Z, Xia Y, Chen Y, Ye X et al. (2020). Gender-Specific Longitudinal Association of Sleep Duration with Blood Pressure among Children: Evidence from CHNS 2004–2015. INT J HYPERTENS.;2020:5475297. 10.1155/2020/5475297. 10.1155/2020/5475297 PMC7374217 32765906

[27] Lee KJ, Carlin JB (2010). Multiple imputation for missing data: fully conditional specification versus multivariate normal imputation. AM J EPIDEMIOL.

[28] Royston P (2009). Multiple imputation of missing values: further update of ice, with an emphasis on categorical variables. Stata J.

[29] Bozbulut R, Ertaş-Öztürk Y, Döğer E, Bideci A, Köksal E (2020). Increased obesity awareness and adherence to Healthy Lifestyle-Diet reduce metabolic syndrome risk in overweight children. J AM COLL NUTR.

[30] Liu Z, Si W, Zhao Q, Tao C. Does subjective Dietary Knowledge Affect Sugar-Sweetened Carbonated Beverages Consumption and child obesity? Empirical evidence from the Inner Mongolia Autonomous Region in China. Int J Environ Res Public Health. 2021;18(7). 10.3390/ijerph18073713. 10.3390/ijerph18073713 PMC8037324 33918208

[31] Oldewage-Theron W, Egal A, Moroka T (2015). Nutrition knowledge and dietary intake of adolescents in Cofimvaba, Eastern Cape, South Africa. ECOL FOOD NUTR.

[32] Velazquez CE, Pasch KE, Ranjit N, Mirchandani G, Hoelscher DM (2011). Are adolescents’ perceptions of dietary practices associated with their dietary behaviors?. J Am Diet Assoc.

[33] Zhen S, Ma Y, Zhao Z, Yang X, Wen D (2018). Dietary pattern is associated with obesity in chinese children and adolescents: data from China Health and Nutrition Survey (CHNS). NUTR J.

[34] Gaspar T, de Matos MG, Luszczynska A, Baban A, Wit J (2014). The impact of a rural or urban context in eating awareness and self-regulation strategies in children and adolescents from eight european countries. INT J PSYCHOL.

[35] Shahsanai A, Farajzadegan Z, Hadi SZ, Heidari K, Omidi R (2018). Assessment of the relationship between Nutritional Knowledge and Anthropometric Indices in Isfahan Children and adolescent. Adv Biomed Res.

[36] Economos CD, Bakun PJ, Herzog JB, Dolan PR, Lynskey VM, Markow D (2014). Children’s perceptions of weight, obesity, nutrition, physical activity and related health and socio-behavioural factors. PUBLIC HEALTH NUTR.

[37] Story M, Neumark-Sztainer D, French S. Individual and environmental influences on adolescent eating behaviors. J Am Diet Assoc. 2002;102(3 Suppl). 10.1016/s0002-8223(02)90421-9. S40-51. 10.1016/s0002-8223(02)90421-9 11902388

[38] Asakura K, Todoriki H, Sasaki S (2017). Relationship between nutrition knowledge and dietary intake among primary school children in Japan: combined effect of children’s and their guardians’ knowledge. J EPIDEMIOL.

[39] Williams L, Campbell K, Abbott G, Crawford D, Ball K (2012). Is maternal nutrition knowledge more strongly associated with the diets of mothers or their school-aged children?. PUBLIC HEALTH NUTR.

[40] Räsänen M, Niinikoski H, Keskinen S, Tuominen J, Simell O, Viikari J (2001). Nutrition knowledge and food intake of seven-year-old children in an atherosclerosis prevention project with onset in infancy: the impact of child-targeted nutrition counselling given to the parents. EUR J CLIN NUTR.

[41] An R, Xiang X, Xu N, Shen J (2020). Influence of Grandparental Child Care on Childhood obesity: a systematic review and Meta-analysis. CHILD OBES.

[42] Sata M, Yamagishi K, Sairenchi T, Ikeda A, Irie F, Watanabe H (2015). Impact of caregiver type for 3-Year-old children on subsequent between-meal eating Habits and being overweight from childhood to Adulthood: a 20-Year follow-up of the Ibaraki Children’s cohort (IBACHIL) study. J EPIDEMIOL.

[43] Guo Y, Yin X, Wu H, Chai X, Yang X (2019). Trends in overweight and obesity among children and adolescents in China from 1991 to 2015: a Meta-analysis. INT J ENV RES PUB HE.

