# Supplementary material for: Influence of adolescents’ and parental dietary knowledge on adolescents’ body mass index (BMI), overweight/obesity in 2004–2015: a longitudinal study
Source: Arch Public Health. 2023 Oct 23;81:188. doi: 10.1186/s13690-023-01197-x (PMC10591379; doi:10.1186/s13690-023-01197-x)
Supplement: Supplementary file 1 — Supplementary Material 1 [file 13690_2023_1197_MOESM1_ESM.docx]

**Supplemental Table 1. Dietary knowledge questions and corresponding correct answers.**

| Do you strongly agree, somewhat agree, somewhat disagree or strongly disagree with this statement? (Options were strongly disagree, disagree, neutral, agree, strongly agree and unknown)  * Please note that the question is not asking about your actual habits. | True/False |
| --- | --- |
| 1. Choosing a diet with a lot of fresh fruits and vegetables is good for one’s health. | T |
| 1. Eating a lot of sugar is good for one’s health. | F |
| 1. Eating a variety of foods is good for one’s health. | T |
| 1. Choosing a diet high in fat is good for one’s health. | F |
| 1. Choosing a diet with a lot of staple foods [rice and rice products and wheat and wheat products] is not good for one’s health. | T |
| 1. Consuming a lot of animal products daily (fish, poultry, eggs and lean meat) is good for one’s health. | F |
| 1. Reducing the amount of fatty meat and animal fat in the diet is good for one’s health. | T |
| 1. Consuming milk and dairy products is good for one’s health. | T |
| 1. Consuming beans and bean products is good for one’s health. | T |
| 1. Physical activities are good for one’s health. | T |
| 1. Sweaty sports or other intense physical activities are not good for one’s health. | T |
| 1. The heavier one’s body is, the healthier he or she is. | F |
| 1. Eating salty foods can cause hypertension. | T |
| 1. Refined grains (rice and wheat flour) contain more vitamins and materials than unrefined grains. | F |
| 1. Lard is healthier than vegetable oils. | F |
| 1. Vegetables contain more starch than staple foods (rice or wheat flour). | F |
| 1. Eggs and milk are the important sources of high-quality protein. | T |
